# Supplementary figures and images for: Two-stage object detection in low-light environments using deep learning image enhancement
Source: PeerJ Comput Sci. 2025 Apr 7;11:e2799. doi: 10.7717/peerj-cs.2799 (PMC12190514; doi:10.7717/peerj-cs.2799)

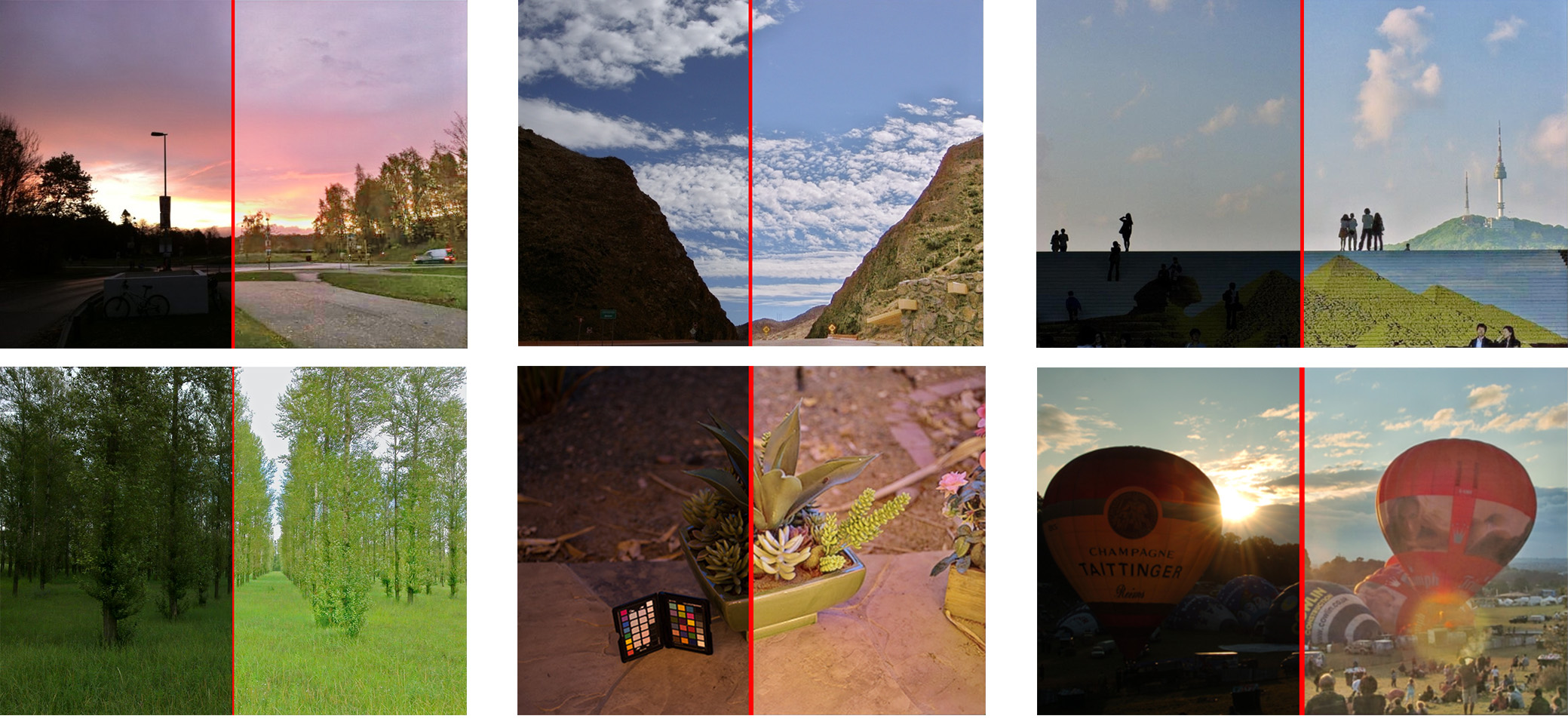

Supplement: Supplemental Information 1 [file peerj-cs-11-2799-s001.zip › ImageEnhancement_TBEFN_Algorithm/TBEFN-master/demo_img/demo_img.jpg]

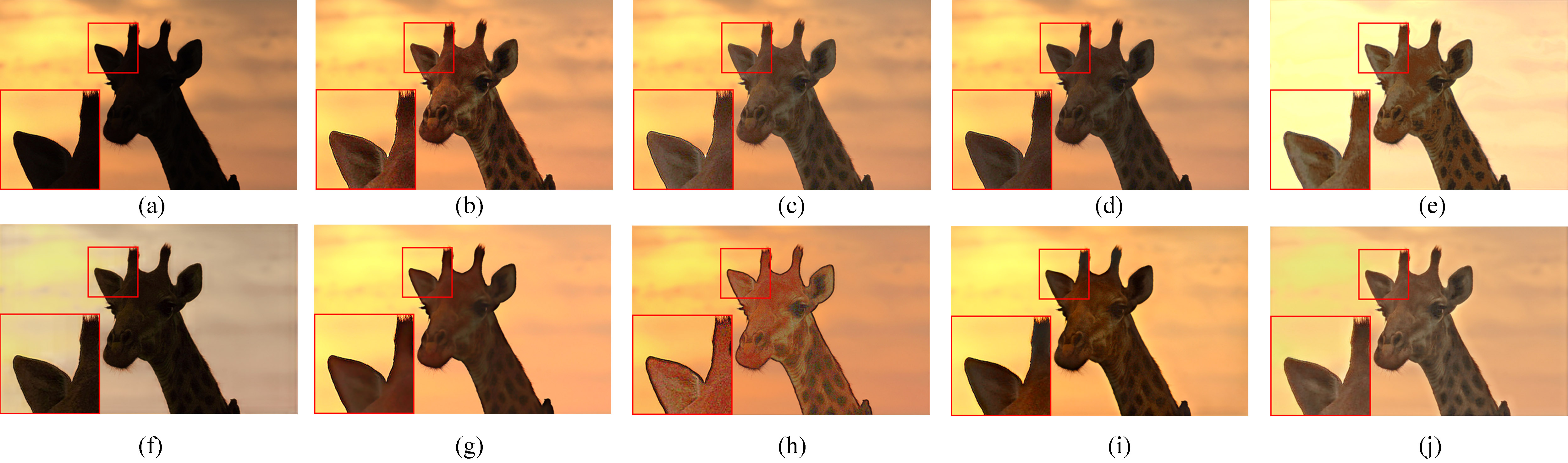

Supplement: Supplemental Information 1 [file peerj-cs-11-2799-s001.zip › ImageEnhancement_TBEFN_Algorithm/TBEFN-master/demo_img/giraffe.jpg]

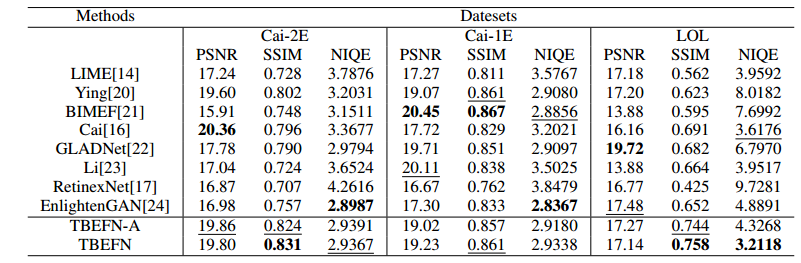

Supplement: Supplemental Information 1 [file peerj-cs-11-2799-s001.zip › ImageEnhancement_TBEFN_Algorithm/TBEFN-master/demo_img/I.png]

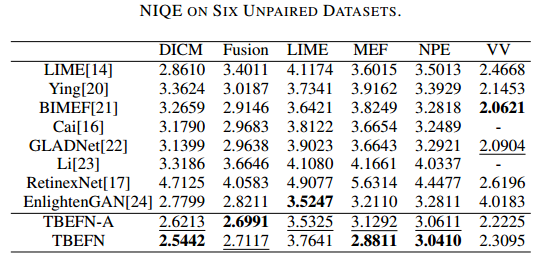

Supplement: Supplemental Information 1 [file peerj-cs-11-2799-s001.zip › ImageEnhancement_TBEFN_Algorithm/TBEFN-master/demo_img/II.png]

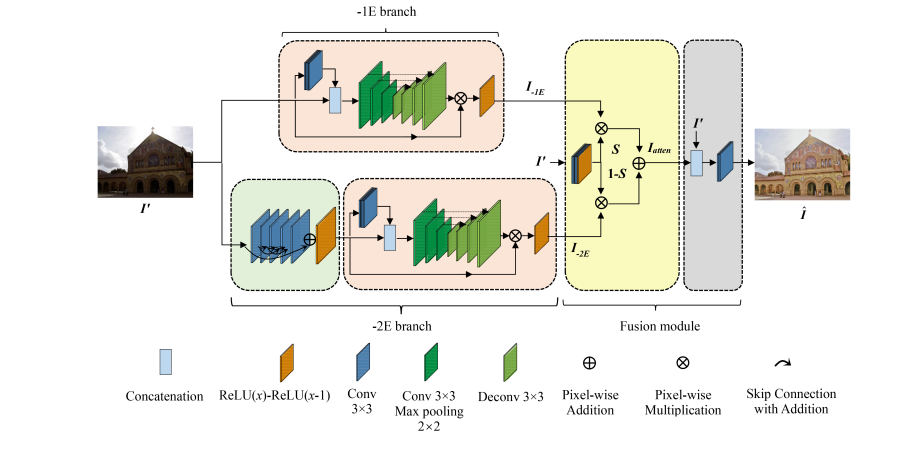

Supplement: Supplemental Information 1 [file peerj-cs-11-2799-s001.zip › ImageEnhancement_TBEFN_Algorithm/TBEFN-master/demo_img/Structure.png]

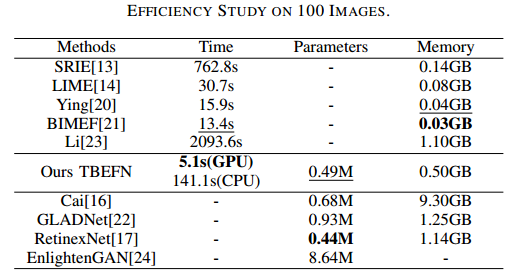

Supplement: Supplemental Information 1 [file peerj-cs-11-2799-s001.zip › ImageEnhancement_TBEFN_Algorithm/TBEFN-master/demo_img/VII.png]

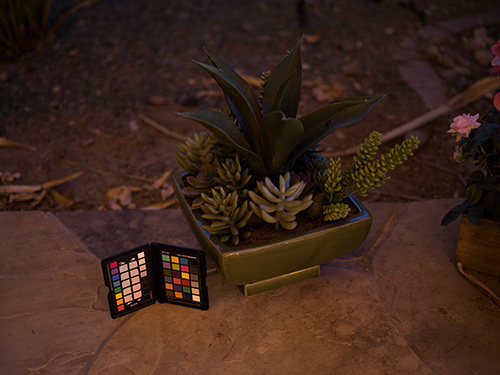

Supplement: Supplemental Information 1 [file peerj-cs-11-2799-s001.zip › ImageEnhancement_TBEFN_Algorithm/TBEFN-master/input_dir/03.bmp]

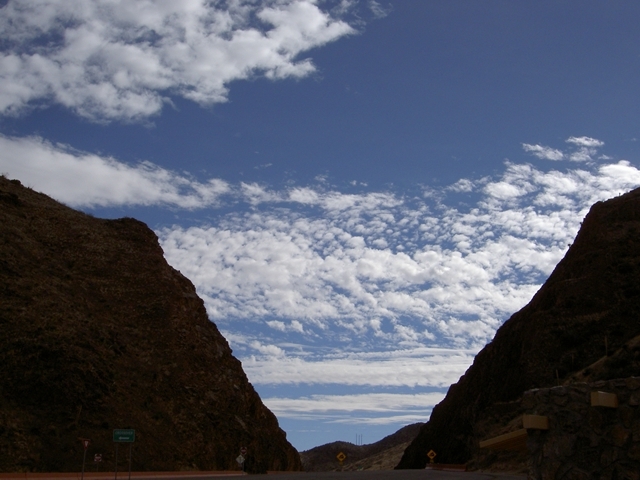

Supplement: Supplemental Information 1 [file peerj-cs-11-2799-s001.zip › ImageEnhancement_TBEFN_Algorithm/TBEFN-master/input_dir/11.JPG]

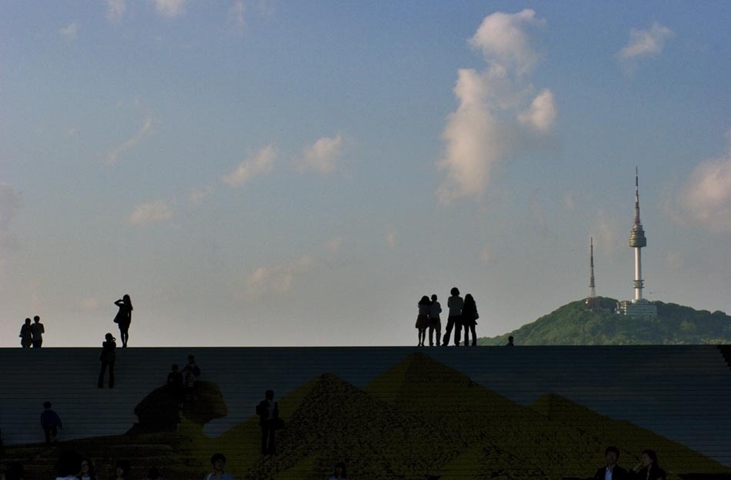

Supplement: Supplemental Information 1 [file peerj-cs-11-2799-s001.zip › ImageEnhancement_TBEFN_Algorithm/TBEFN-master/input_dir/41.jpg]

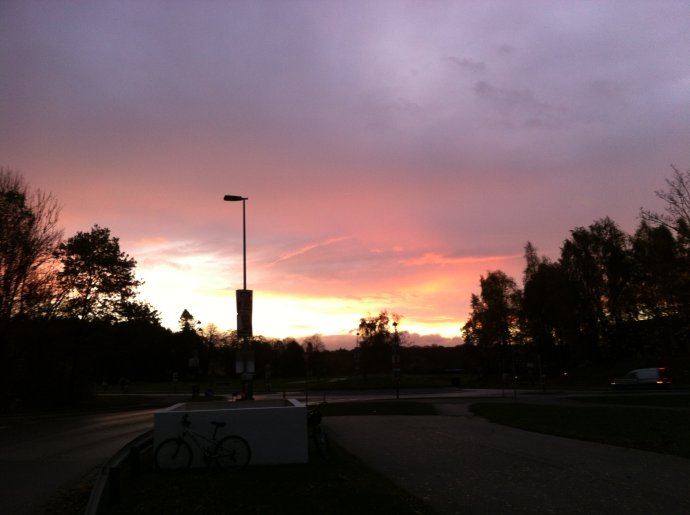

Supplement: Supplemental Information 1 [file peerj-cs-11-2799-s001.zip › ImageEnhancement_TBEFN_Algorithm/TBEFN-master/input_dir/9.jpg]

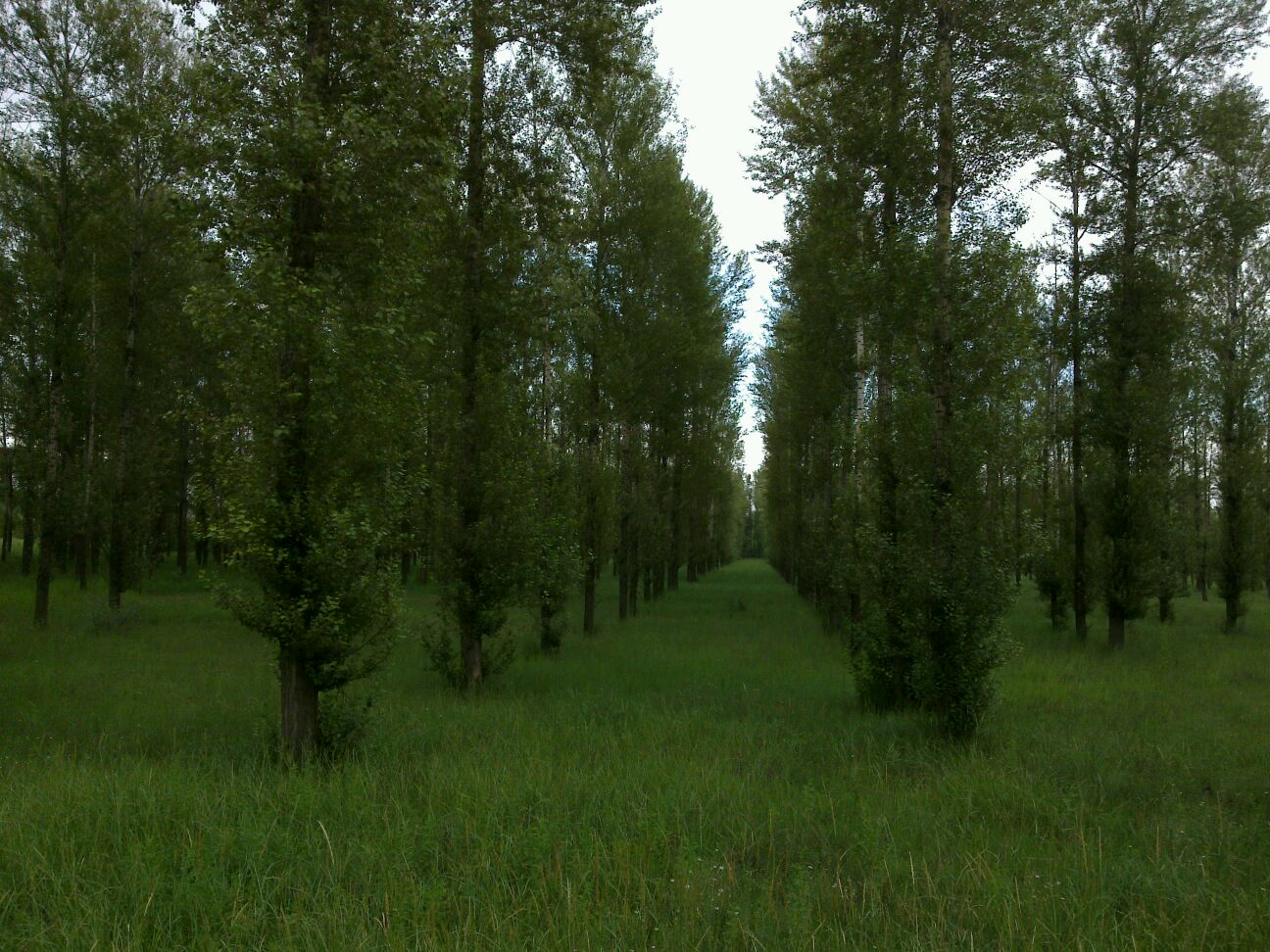

Supplement: Supplemental Information 1 [file peerj-cs-11-2799-s001.zip › ImageEnhancement_TBEFN_Algorithm/TBEFN-master/input_dir/cloudy (10)_bmp.bmp]

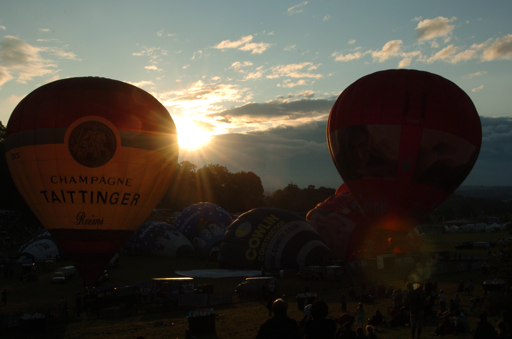

Supplement: Supplemental Information 1 [file peerj-cs-11-2799-s001.zip › ImageEnhancement_TBEFN_Algorithm/TBEFN-master/input_dir/DSC_0168.png]

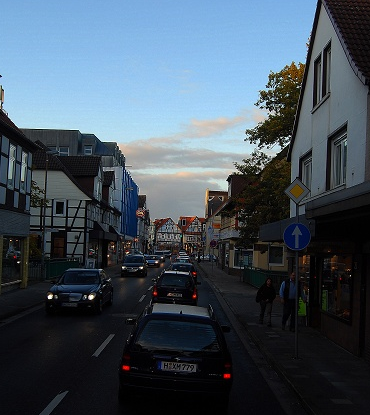

Supplement: Supplemental Information 3 [file peerj-cs-11-2799-s003.zip › GLADNet-master/data/eval/low/4.bmp]

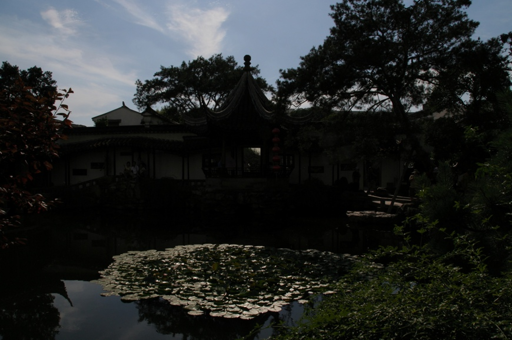

Supplement: Supplemental Information 3 [file peerj-cs-11-2799-s003.zip › GLADNet-master/data/eval/low/chinese_garden3.png]

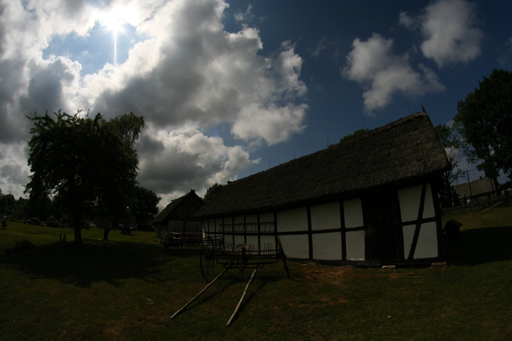

Supplement: Supplemental Information 3 [file peerj-cs-11-2799-s003.zip › GLADNet-master/data/eval/low/kluki1.png]
